# Age-related knowledge deficit and attitudes towards oral implants: Survey-based examination of the correlation between patient age and implant therapy awareness

**DOI:** 10.1186/s12903-024-04134-8

**Published:** 2024-03-29

**Authors:** Ina Nitschke, Kristina Krüger, Julia Jockusch

**Affiliations:** 1 Clinic of General, Special Care and Geriatric Dentistry, Center of Dental Medicine, University of Zurich, Zurich, 8032 Switzerland; 2 Department of Prosthodontics and Materials Science, Gerodontology Section, University of Leipzig, 04103 Leipzig, Germany; 3 University Research Priority Program “Dynamics of Healthy Aging”, University of Zurich, Stampfenbachstrasse, Zurich, CH-8006 Switzerland

**Keywords:** Dental implants, Oral implants, Old age, Awareness, Knowledge, Attitudes, Patients’ perspective

## Abstract

**Background:**

Implantology, as a recognized therapeutic approach, is gaining prominence. The decision-making process and success of implant therapy are closely linked to patient knowledge and expectations. This study aims to explore the association between age and knowledge regarding oral implants.

**Methods:**

Participants were categorized into three age groups (ag): ag 1 (35–44 years), ag 2 (65–74 years), and ag 3 (75 years and older). A total of 400 participants per age group were randomly selected using data from the residents’ registration office of Berlin, Germany. Structured telephone interviews were conducted between 2016 and 2017, employing a 67-item questionnaire covering awareness, information level, cost estimation, attitudes, and experiences with oral implants.

**Results:**

Despite a low overall knowledge level across all age groups, there was no significant correlation between age and knowledge about oral implants. Awareness increased with age. Information sources varied, with friends, acquaintances, and dentists playing key roles. Participants expressed diverse opinions on implants, with durability and stability identified as crucial characteristics. Significant differences in knowledge were observed between age groups regarding awareness, information sources, and perceptions of dentists offering implants.

**Conclusions:**

The study suggests a need for targeted educational programs, emphasizing age-appropriate information sources to enhance health literacy in oral implantology, particularly among older individuals. Educating physicians on oral implant basics is also crucial. Implementing these measures could empower individuals to make informed decisions about oral implant treatment, thereby contributing to improved oral health outcomes.

## Background

As a scientifically recognized therapy, implantology is gaining increasing importance [1]. It plays an integral role in the prosthetic rehabilitation of patients as part of comprehensive dental treatment [2]. Consequently, the number of patients benefiting from implant treatment is also increasing, thanks to advancements in augmentation techniques and implant surfaces [3]. Successful insertion and osseointegration of oral implants are now possible even in the presence of local bone defects, reducing the significance of contraindications related to bone morphology [4].

Despite systemic, local, or patient-related contraindications, oral placement of implants can be feasible under general medical supervision, after appropriate pretreatment, and with peri- and postoperative measures [5]. The number of dentists offering implant services in their practices is also on the rise [6]. Restorations supported with oral implants are increasingly used to replace missing teeth; which is sometimes considered by implantologists to be the new gold standard [7]. However, multimorbidity in old age and physical resources necessitate a critical examination of the provision of fixed and removable dental prostheses [8]. Additionally, implant therapy can be considered a safe procedure in some patients suffering from systemic diseases, such as compensated diabetes [9]. Older patients may also have other systemic impairments, such as the intake of bisphosphonates or other medications associated with osteonecrosis. This must be considered before implantation. Research has shown that there is a trend towards the occurrence of medication-related osteonecrosis of the jaw (MRONJ) as a complication of dentoalveolar surgery, which proportionally mostly affects males and the posterior mandibular sectors [10].

While scientific studies primarily focus on the clinical aspects of oral implantology such as osseointegration, survival rates [11], biological and mechanical complications [12], and patient satisfaction [13], empirical data on the general population’s perception and knowledge of oral implants are scarce [14–16]. Moreover, older adults and their heterogeneity are underrepresented in studies on implant dentistry [17]. Only a few studies have looked more closely at awareness, knowledge, and acceptance in the field of implant dentistry. A significant proportion of the population is often poorly or misinformed [18]. There appears to be considerable variation in the population in terms of assumed versus real knowledge about the cost, survival rates, and appropriate follow-up needs of oral implants [18].

Given that the decision for implant therapy and its success significantly correlate with patients’ knowledge and expectations [19], the aim of this study is to specify this knowledge and define a potential correlation with age. We hypothesize that the level of knowledge about oral implants is low in all age groups. Furthermore, we hypothesize that with increasing age, awareness, knowledge, and acceptance of oral implants decrease, thus suggesting an age-related knowledge gap in this area.

## Methods

### Participant

Participants were selected in three age groups (ag): ag 1: 35–44 years, ag 2: 65–74 years, ag 3: 75 years and older. Randomized data sets of 200 potential participants per age group were requested from the residents’ registration office of the city of Berlin, the capital of Germany. Telephone numbers were obtained based on the addresses. Younger people often do not have landline telephones but mobile phones. In Germany, mobile telephone numbers cannot be determined from the registration address in the context of scientific studies (data protection/privacy). Therefore, an additional set with the same number of randomized records was requested from the residents’ registration office of the city of Berlin. This resulted in a total number of *n* = 400 participants per age group. The structured telephone interviews were conducted as part of a telephone survey between 2016 and 2017. Interview refusers were replaced by statistical twins who had the same age characteristics. The assumption is that statistical twins also have commonalities in non-quantified characteristics. This approach counteracts the visible sample shrinkage in quota samples. [19] Apart from age and the presence of a telephone number/telephone connection only one further inclusion/exclusion criteria was defined: all participants had to be proficient in the German language.

### Data collection instrument

The structured questionnaire (67 items) included both open-ended questions (free response options) and closed-ended questions with a predetermined rating scale consisting of seven response options. Multiple responses were allowed in some cases. The 7-point Likert scale was used for conducting relevant statistical procedures [20]. To avoid potential bias, the questions were formulated in simple language, clearly and neutrally.

Initially, participants were informed about the objective of the interview. Subsequently, their consent for the processing of their data was obtained. The voluntary nature of study participation was emphasized, and the preservation of anonymity during data analysis was assured. A brief instruction on how to answer the questions was provided. At the beginning of the interview, information about dental visit behavior was documented. Did the study participant have a regular dentist? How often was the dentist visited in the last 12 months? What was the reason for the last dental visit?

Data on awareness of implants, level of information, estimated cost, personal attitudes, and experiences with oral implants were then collected. Participants were asked if they had ever heard of the treatment with oral implants before the interview, whether anyone in their circle of friends or acquaintances had an oral implant, and how satisfied that person was with it.

Participants were then asked to indicate how well-informed they felt about oral implants and other options for dental prosthetics. Response options included very well, well, partly/partly, little, not at all, no response, and don’t know. Participants were then asked to estimate the cost of such an implant, including the superstructure and laboratory cost. To assess personal attitudes and experiences, it was documented whether participants would consider getting an oral implant to replace missing teeth or why treatment with artificial tooth roots was not considered. Furthermore, participants were asked to describe their general opinion on oral implants. Response options included implants are expensive, if necessary, I would consider them, implants are not suitable for everyone, implants are suitable for everyone, I would rather not get them, everyone can afford implants, no response, don’t know.

The collection of knowledge in the field of oral implantology, followed by the naming of the advantages and disadvantages of implant therapy, constituted the essential part of the interview. This section included questions about materials, anchoring possibilities, lifespan of dental implants, a possible age limit for treatment, and the need for special care and hygiene measures for oral implants. In addition, participants were asked to name the characteristics of oral implants and rank their importance. Socio-demographic questions regarding age, gender, and educational level were asked at the end.

The questionnaire was developed specifically for this study and checked in advance on some participants as part of a pilot test concerning comprehensibility etc. Validity and reliability were not tested. All interviews were conducted by one examiner.

### Statistical considerations

Participant data were anonymized using an identification number and recorded in a database. Data collection and analysis were performed using SPSS Version 27.0 (SPSS Statistics, IBM, Chicago, Illinois, USA).

Quantitative features, such as the estimated cost of an implant with a superstructure including necessary laboratory cost, were presented by calculating means and creating a cross-tabulation. Post-hoc tests for multiple comparisons were then conducted to determine significant differences between means. Additionally, this was also examined using a one-way analysis of variance (ANOVA). The Levene test was performed to assess the homogeneity of variances among the defined age groups as respective populations. The Pearson chi-square test was used to demonstrate the distribution of the collected data and examine the relationship between two nominal-scaled variables, such as the participant’s age and their knowledge of oral implants regarding material and longevity. Histograms and cross-tabulations were used for graphical representation. A *p*-value of < 0.05 was defined as statistically significant.

## Results

Out of a total of 1200 potential participants (*n* = 400 per age group (ag)), 159 participants were included in the analysis (response rate: ag 1: 12.5%; ag 2: 14.5%; ag 3: 12.8%). In all three ag (mean (± SD): ag 1: 39.6 ± 3.3 years; ag 2: 68.7 ± 3.1 years; ag 3: 81.4 ± 5.1 years), the proportion of women predominated (ag 1 (*n* = 50): female *n* = 27, 54%; ag 2 (*n* = 58): female *n* = 35, 60.3%; ag 3 (*n* = 51): female *n* = 31, 60.8%).

Study participation was declined for similar reasons in all three ag (*n* = 626). Lack of interest (*n* = 122) and lack of time (*n* = 156) were the most cited reasons. In ag 3, however, the lack of understanding of the terminology and communication over the phone, e.g., hearing impairments was also relevant. Doubt and shame of providing false information also played a role in this ag.

### Awareness, level of information, and opinions regarding oral implants and alternative treatment methods

Awareness of oral implants as a possible treatment option varied significantly between the ag (ANOVA *p* = 0.011, Bonferroni correction ag 1:ag 2 *p* = 0.014), awareness increases with age. Almost all participants in all ag had never heard of guided or immediate placement of implants (Table 1).

**Fig. 1:**
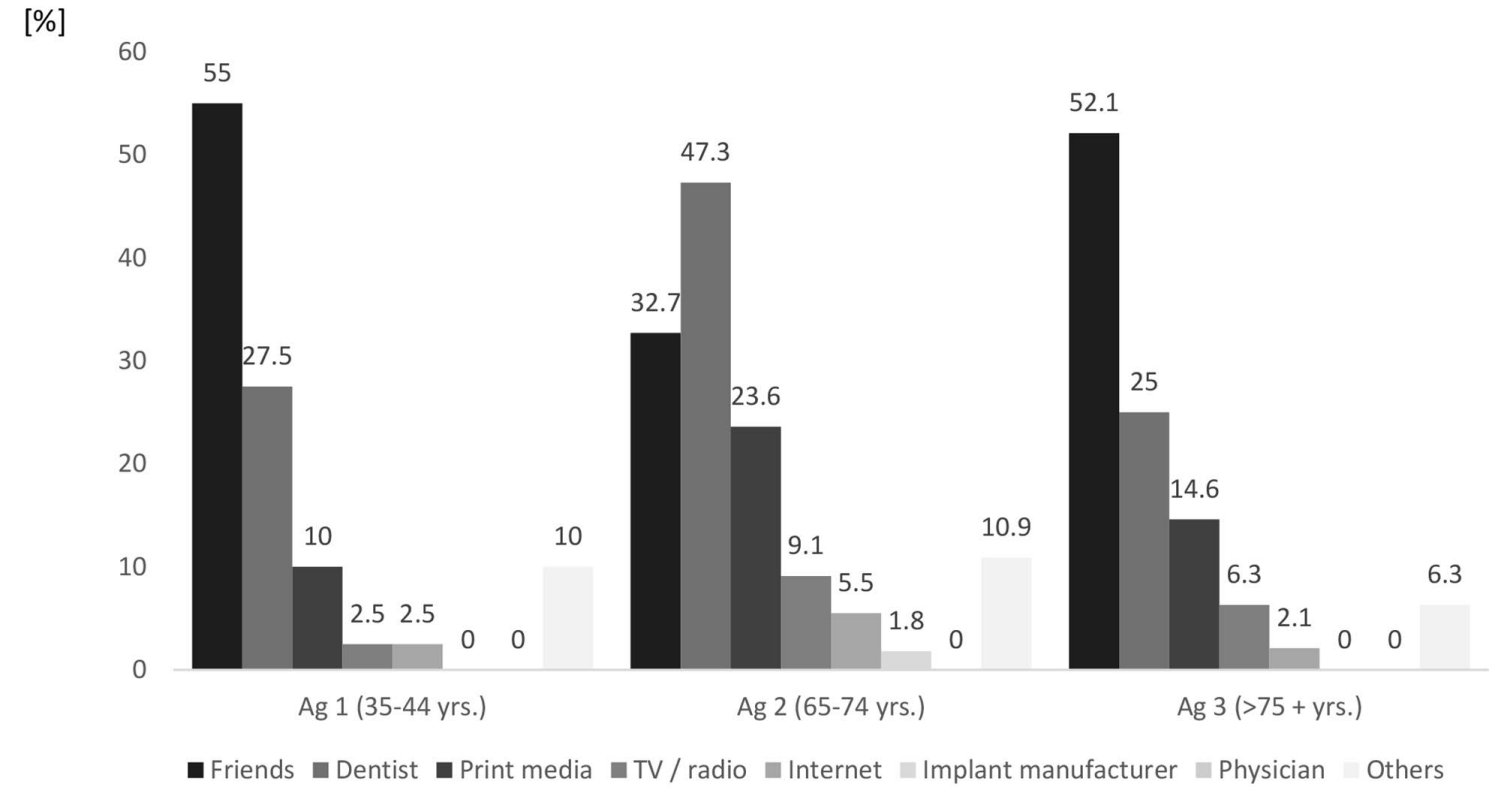
Sources of information on implants used by participants, separated by age group (Ag). Multiple responses were possible (*n* = 159)

More than half of all participants in ag 1 and ag 3 obtained their information about implants from friends and acquaintances, while less than one-third of participants in these two ag obtained the information from their dentist. In ag 2, however, information was obtained almost equally from the dentist, friends, and acquaintances, and print media. No one identified their physician as a source of information. Implant manufacturers were exclusively used as an information source by participants in ag 2 (Fig. 1).

14.0% of ag 1 participants felt very well to well informed, while 64.0% felt little to not informed at all about oral implants. One-fifth of ag 1 participants felt partially informed. In ag 2, 51.8% reported feeling very well to well informed, and 16.1% felt little to not informed at all. About one-third (32.1%) of ag 2 participants felt only partially informed. In ag 3, 25.0% felt very well to well informed, 46.2% felt little to not informed at all, and almost one-third (28.8%) felt only partially informed. Therefore, there is no difference in the level of information among the defined age groups.

Especially participants in ag 2 feel predominantly very well or well informed about alternative options for dental prosthetics without implants (60.7%), while 64.0% of ag 1 participants and 33.3% of ag 3 participants feel little to not informed about alternative treatment options.

Regarding the question of whether participants knew whether their own dentist offers implants as a treatment alternative, there was a significant difference between ag 1 and ag 2 participants (ANOVA *p* = 0.006, Bonferroni correction ag 1:ag 2 *p* = 0.004). Ag 2 participants were the most informed about whether their dentist offers oral implants or not, while nearly half of ag 1 participants did not know (Table 1).

**Fig. 2:**
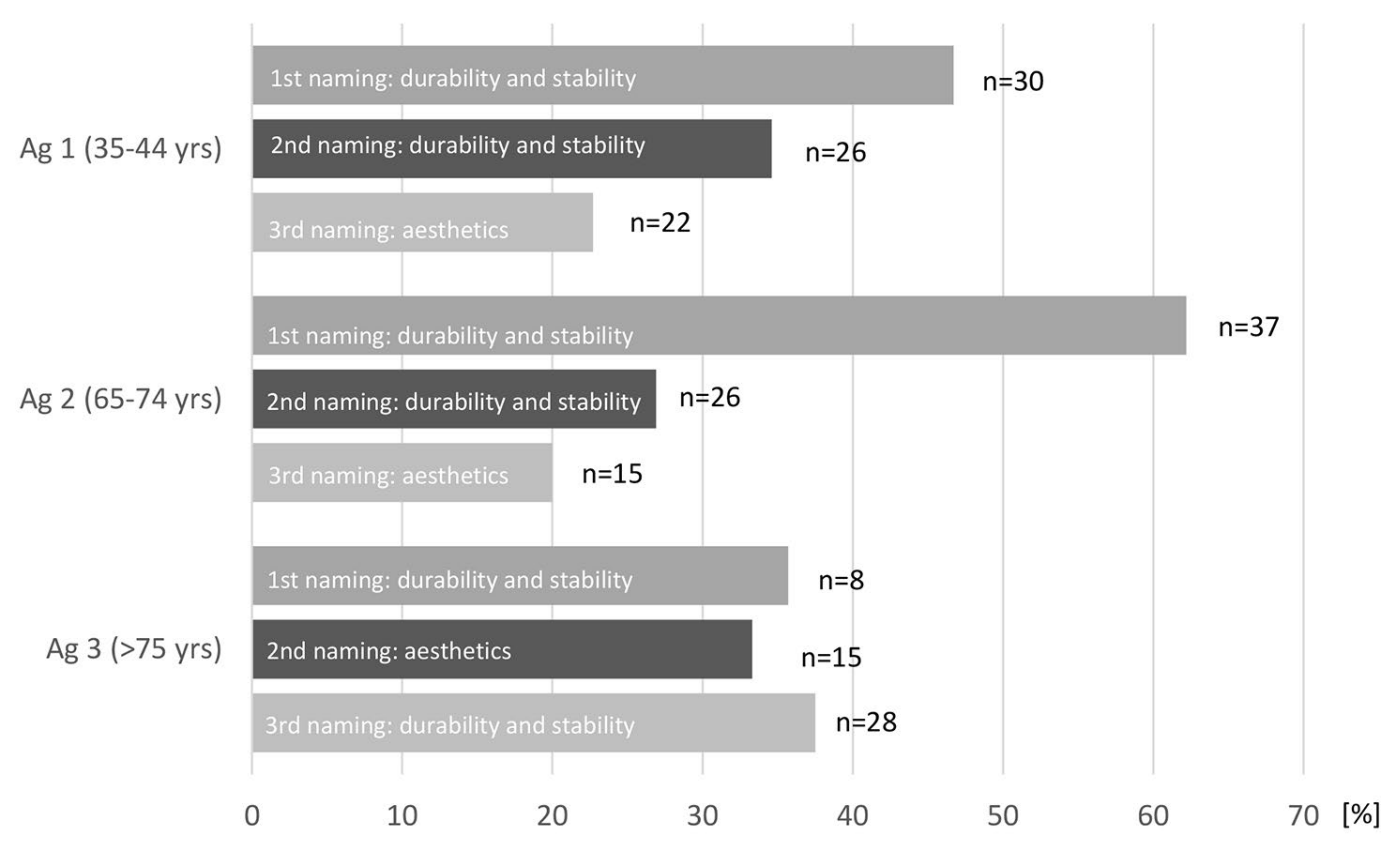
Mention of the three most important characteristics of implants separated by age group (Ag)

When asked about the most important characteristic of implants, participants in all ag most frequently mentioned “durability and stability” as the first response. As the second most important characteristic, participants in ag 1 and ag 2 again mentioned “durability and stability”. In ag 3, the second most frequent response was “aesthetics”. In ag 1 and ag 2, the third most important characteristic was also “aesthetics”, while in ag 3, the third most frequent response was “durability and stability” (Fig. 2).

With increasing age, the proportion of participants who have no interest in further information about implants significantly increases (Pearson Chi2-test *p* < 0.001). If further information is desired, the dentist is the most frequently mentioned desired source of information in all age groups (Table 1).

**Table 1:** Aspects of awareness, information level, and opinions regarding oral implants separated by gender (female *n* = 93, male *n* = 66) and age group (ag 1 *n* = 50, ag 2 *n* = 57, ag 3 *n* = 52), (Total (all participants) *n* = 159, n/% - number/percent, Ag – age groups; bold values in the “p” column indicate statistical significance (p – ANOVA and Bonferoni) with a significance level of *p* < 0.05), * purely descriptive analysis

	Total	Sex			Age group			
	All *n* = 159 [n/%]	Female *n* = 93 [n/%]	Male *n* = 66 [n/%]	*p*	Ag 1 (35–44 yrs) *n* = 50 [n/%]	Ag 2 (65–74 yrs) *n* = 57 [n/%]	Ag 3 (> 75 yrs) *n* = 52 [n/%]	*p*
Have you ever heard about a treatment with oral implants, which are artificial tooth roots, before our conversation?								
	*n* = 159	*n* = 93	*n* = 66	0.626	*n* = 50	*n* = 57	*n* = 52	**0.011**
No Yes	16 / 10.1 143 / 89.9	10 / 10.8 83 / 89.2	6 / 9.1 60 / 90.9		10 / 20.0 40 / 80.0	2 / 3.5 55 / 96.5	4 / 7.7 48 / 92.3	**Bonferoni** **ag1:ag2 0.014**
Have you heard/read anything about computer-guided, navigated placement of implants in the past 5 years (recent past)?								
	*n* = 159	*n* = 93	*n* = 66	0.340	*n* = 50	*n* = 57	*n* = 52	0.323
No Yes	142 / 89.3 17 / 10.7	81 / 87.1 12 / 12.9	61 / 92.4 5 / 7.6		47 / 94.0 3 / 6.0	48 / 84.2 9 / 15.8	47 / 90.4 5 / 9.6	
Have you heard/read about immediate placement of implants?								
	*n* = 159	*n* = 93	*n* = 66	0.203	*n* = 50	*n* = 57	*n* = 52	0.274
No Yes	135 / 84.9 24 / 15.1	76 / 81.7 17 / 18.3	59 / 89.4 7 / 10.6		45 / 90.0 5 / 10.0	45 / 78.9 12 / 21.1	45 / 86.5 7 / 13.5	
Do you know if your dentist offers oral implants as a treatment alternative?								
	*n* = 148	*n* = 86	*n* = 62	0.765	*n* = 48	*n* = 55	*n* = 45	**0.006**
Yes, she/he offers. No, she/he does not offer. I don’t know.	92 / 62.2 10 / 6.7 46 / 31.1	55 / 63.9 6 / 7.0 25 / 29.1	37 / 59.7 4 / 6.5 21 / 33.8		23 / 47.9 3 / 6.3 22 / 45.8	41 / 74.5 4 / 7.3 10 / 18.2	28 / 62.2 3 / 6.7 14 / 31.1	**Bonferoni** **ag1:ag2 0.004**
Would you be interested in receiving more information about oral implants?								
	*n* = 157	*n* = 93	*n* = 64	0.181	*n* = 49	*n* = 57	*n* = 51	0.140
No Yes	107 / 68.2 50 / 31.8	63 / 67.7 30 / 32.3	44 / 66.7 20 / 30.3		25 / 51.0 24 / 49.0	36 / 63.2 21 / 36.8	46 / 90.2 5 / 9.8	
Where would you prefer to obtain the additional information from?								
	*n* = 68	*n* = 40	*n* = 28	*	*n* = 32	*n* = 30	*n* = 6	*
Dentist	41 / 60.3	24 / 60.0	17 / 60.7		21 / 65.6	16 / 53.3	4 / 66.6	
Other	11 / 16.2	5 / 12.5	6 / 21.4		3 / 9.4	8 / 26.6	0 / 0	
Friends/acquaintances	8 / 11.8	5 / 12.5	3 / 10.7		6 / 18.8	1 / 3.4	1 / 16.7	
Print media	7 / 10.3	5 / 12.5	2 / 7.2		2 / 6.2	4 / 13.3	1 / 16.7	
I don’t know.	1 / 1.4	1 / 2.5	0 / 0		0 / 0	1 / 3.4	0 / 0	
General practitioner	0 / 0	0 / 0	0 / 0		0 / 0	0 / 0	0 / 0	
Implant manufacturers	0 / 0	0 / 0	0 / 0		0 / 0	0 / 0	0 / 0	

### Knowledge about oral implants

Titanium as a material was most mentioned by participants in ag 2 (*n* = 16, 33.3%), while ceramic was mentioned most by participants in ag 1 (*n* = 14, 23.3%) and ag 2 (*n* = 7, 25.0%). Approximately one-quarter of all participants in all ag stated that implants are made of ceramic (Table 2).

Most participants in all ag knew that implants are anchored in the bone (ag 1: *n* = 39, 78.0%; ag 2: *n* = 50, 87.7%; ag 3: *n* = 39, 75.0%). (Table 2) and believe that implants require the same or more care as natural teeth (Table 2).

Regarding the question of whether there is an age limit for receiving implants, there is a significant difference between the ag (Pearson Chi 2 test *p* = 0.028). Participants in ag 1 and ag 2 most commonly believe there is no age limit (ag 1: *n* = 33, 66.0%; ag 2: *n* = 28, 49.1%), while participants in ag 3 most commonly do not know if there is an age restriction (*n* = 24, 48.0%) (Table 2).

There was no difference between ag in the estimation of how long an oral implant would typically last (Median (Range): ag 1: 10 (3–20) years; ag 2: 12 (5–30) years; ag 3: 12.5 (5–20) years) (Table 2).

### Financing of oral implants

Most participants in all ag assume that they themselves as patients (ag 1: *n* = 22, 45.8%; ag 2: *n* = 31, 56.4%; ag 3: *n* = 27, 51.9%) or in combination with the health insurance (ag 1: *n* = 18, 37.5%; ag 2: *n* = 18, 32.7%; ag 3: *n* = 16, 30.8%) would bear the cost of an implant. The cost for an implant including a crown is reported in the median (range) as 2000 Euros (range 300–8000 Euros) across all ag, with participants in ag 1 indicating a slightly higher median price for an implant including a crown (Median Implant and Crown 2750 Euros ((Range) 600–8000 Euros)) compared to participants in the other two ag (Table 3).

### Opinions and assessment of the advantages and disadvantages of oral implants

When asked about their attitudes towards implants, the participants in ag 1 (*n* = 68, multiple answers possible) mainly stated that implants are expensive for them (*n* = 20, 29.4%). However, they would have implants inserted if necessary (*n* = 19, 27.9%). Most participants in ag 2 (*n* = 79, multiple responses possible) stated that they would have implants if needed (*n* = 22, 27.8%), and the second most common response was that implants are expensive (*n* = 14, 17.7%). In ag 3 (*n* = 79, multiple responses possible), there are more participants who would not have implants (*n* = 26, 32.9%) than those who would have them implanted if needed (*n* = 14, 17.7%). The cost factor is also mentioned here (*n* = 17, 21.5%) (Table 4).

When asked about the advantages of implants, it was found that the importance of aesthetic aspects plays a significantly lesser role with increasing age (Pearson Chi 2 test *p* = 0.033). Participants in ag 1 (*n* = 93, multiple answers possible) mentioned aesthetics (*n* = 20, 21.5%) as well as functionality (*n* = 12, 12.9%), achieving higher bite forces (*n* = 11, 11.8%), and their longer durability (*n* = 10, 10.8%) as advantages of implants. Participants in ag 2 (*n* = 91, multiple answers possible) primarily mentioned advantages in aesthetics (*n* = 14, 15.4%) and the fact that reconstructions with implants do not feel like removable dentures (*n* = 14, 15.4%). Participants in ag 3 (*n* = 77, multiple answers possible) mainly mentioned the advantage that reconstructions with implants do not feel like removable dentures (*n* = 12, 15.6%).

There were no significant differences between ag regarding the mentioned disadvantages of implants. For most participants in ag 1 (*n* = 71, multiple answers possible), the disadvantages lie in the high cost (*n* = 29, 40.8%), for most participants in ag 2 (*n* = 87, multiple answers possible), it is the high cost (*n* = 22, 25.3%) and possible complications (*n* = 20, 23.0%), and for participants in ag 3 (*n* = 71, multiple answers possible), the high cost is disadvantageous (*n* = 20, 28.2%) (Table 4).

**Table 2:** Knowledge about oral implants separated by gender (female *n* = 93, male *n* = 66) and age group (ag 1 *n* = 50, ag 2 *n* = 57, ag 3 *n* = 52), (Total (all participants) *n* = 159, n/% - number/percent, Ag – age groups; bold values in the “p” column indicate statistical significance (p – ANOVA and Bonferoni) with a significance level of *p* < 0.05), * purely descriptive analysis

	Total	Sex			Age group			
	All *n* = 159 [n/%]	Female *n* = 93 [n/%]	Male *n* = 66 [n/%]	*p*	Ag 1 (35–44 yrs) *n* = 50 [n/%]	Ag 2 (65–74 yrs) *n* = 57 [n/%]	Ag 3 (> 75 yrs) *n* = 52 [n/%]	*p*
What material are implants made of?								
	*n* = 136	*n* = 78	*n* = 58	Not possible	*n* = 60	*n* = 48	*n* = 28	Not possible
Titanium	35 / 25.7	23 / 29.5	12 / 20.7		13 / 21.7	16 / 33.3	6 / 21.4	
Ceramic	33 / 24.3	16 / 20.5	17 / 29.3		14 / 23.3	12 / 25.0	7 / 25.0	
Steel	25 / 18.4	12 / 15.4	13 / 22.4		12 / 20.0	9 / 18.8	4 / 14.3	
Plastic	22 / 16.2	13 / 16.7	9 / 15.5		13 / 21.7	5 / 10.4	4 / 14,3	
Others	12 / 8.8	9 / 11.5	3 / 5.2		3 / 5.0	6/ 12.5	3/ 10.7	
Gold	9 / 6.6	5 / 6.4	4 / 6.9		5 / 8.3	0 / 0	4 / 14.3	
Where are oral implants anchored?								
	*n* = 159	*n* = 93	*n* = 66	Not possible	*n* = 50	*n* = 57	*n* = 52	Not possible
In the jawbone	128 / 80.5	75 / 80.6	53 / 80.3		39 / 78.0	50 / 87.7	39 / 75.0	
I don’t know.	21 / 13.2	13 / 14.0	8 / 12.1		5 / 10.0	3 / 5.3	13 / 25.0	
To neighboring teeth	6 / 3.8	2 / 2.2	4 / 6.1		4 / 8.0	2 / 3.5	0 / 0	
In the gum tissue	4 / 2.5	3 / 3.2	1 / 1.5		2 / 4.0	2 / 3.5	0 / 0	
How long does an oral implant typically last? (in years)								
	*n* = 65	*n* = 34	*n* = 31	**0.031**	*n* = 22	*n* = 29	*n* = 14	0.56
Mean ± SD Median (Range)	13.1 ± 6.1 12 (3–30)	15 ± 6.9 12.5 (5–30	10.9 ± 4.2 10 (3–20)		12.6 ± 5.5 10 (3–20)	14 ± 7.2 12 (5–30)	11.9 ± 3.9 12.5 (5–20)	4
Are special care and hygiene measures necessary when having oral implants?								
	*n* = 157	*n* = 92	*n* = 65	Not	*n* = 50	*n* = 56	*n* = 51	Not possible
No, they require the same care as natural teeth. No, they require less care than natural teeth. Yes, they require more care than natural teeth. I don’t know.	80 / 51.0 5 / 3.2 47 / 29.9 25 / 15.9	49 / 53.3 0 / 0 28 / 30.4 15 / 16.3	31 / 47.7 5 / 7.7 19 / 29.2 10 / 15.4	possible	22 / 44.0 3 / 6.0 20 / 40.0 5 / 10.0	32 / 57.1 1 / 1.8 17 / 30.4 6 / 10.7	26 / 51.0 1 / 2.0 10 / 19.6 14 / 27.5	
Is there an age limit for patients to receive oral implants?								
	*n* = 157	*n* = 91	*n* = 66	0.782	*n* = 50	*n* = 57	*n* = 50	**0.024** **Bonferoni** **ag1:ag3 0.015**
No Yes I don`t know.	82 / 52.2 23 / 14.6 52 / 33.1	43 / 47.3 17 / 18.7 31 / 34.1	39 / 59.1 6 / 9.1 21 / 31.8		33 / 66.0 6 / 12.0 11 / 22.0	28 / 49.1 12 / 21.1 17 / 29.8	21 / 42.0 5/ 10.0 24 / 48.0	

**Table 3:** Opinions about financing of implants by gender (female *n* = 93, male *n* = 66) and age group (ag 1 *n* = 50, ag 2 *n* = 57, ag 3 *n* = 52), (Total (all participants) *n* = 159, n/% - number/percent, Ag – age groups

	Total	Sex		Age group		
	All *n* = 159 [n/%]	Female *n* = 93 [n/%]	Male *n* = 66 [n/%]	Ag 1 (35–44 yrs) *n* = 50 [n/%]	Ag 2 (65–74 yrs) *n* = 57 [n/%]	Ag 3 (> 75 yrs) *n* = 52 [n/%]
Who bears the cost for an oral implant?						
	*n* = 155	*n* = 93	*n* = 62	*n* = 48	*n* = 55	*n* = 52
Patient themselves	80 / 51.6	52 / 55.9	28 / 45.2	22 / 45.8	31 / 56.4	27 / 51.9
Patient & Health insurance	52 / 33.5	29 / 31.2	23 / 37.1	18 / 37.5	18 / 32.7	16 / 30.8
I don’t know.	11 / 7.1	7 / 7.5	4 / 6.5	3 / 6.3	2 / 3.6	6 / 11.5
Private insurance/supplementary insurance	10 / 6.5	5. / 5.4	5 / 8.1	4 / 8.3	4 / 7.3	2 / 3.8
Health insurance	2 / 1.3	0 / 0	2 / 3.2	1 / 2.1	0 / 0	1 / 1.9
Who should bear the cost for an oral implant?						
	*n* = 155	*n* = 89	*n* = 66	*n* = 49	*n* = 55	*n* = 51
Social insurance/health insurance	84 / 54.2	46 / 51.7	38 / 57.6	31 / 63.3	31 / 56.4	22 / 43.1
Patient & social insurance/health insurance	39 / 25.2	24 / 27.0	15 / 22.7	11 / 22.4	17 / 30.9	11 / 21.6
I don’t know.	17 / 11.0	11 / 12.4	6 / 9.1	2 / 4.1	4 / 7.3	11 / 21.6
Patient themselves	12 / 7.7	7 / 7.9	5 / 7.6	4 / 8.2	2 / 3.6	6 / 11.8
Private insurance/supplementary insurance	3 / 1.9	1 / 1.1	2 / 3.0	1 / 2.0	1 / 1.8	1 / 2.0
Please estimate how much an implant with a replacement crown (including laboratory cost) would cost at the dentist?						
	*n* = 126	*n* = 70	*n* = 56	*n* = 44	*n* = 49	*n* = 33
Mean ± SD in Euro Median (Range) in Euro	2492 ± 1695 2000 (300–8000)	2391 ± 1470 2000 (300–8000)	2619 ± 1946 2000 (300–8000)	2968 ± 1850 2750 (600–8000)	2285 ±1499 2000 (300–8000)	2167 ± 1661 2000 (300–7500)

**Table 4:** Opinions and assessment of the advantages and disadvantages of implants by gender (female *n* = 93, male *n* = 66) and age group (ag 1 *n* = 50, ag 2 *n* = 57, ag 3 *n* = 52), (Total (all participants) *n* = 159, n/% - number/percent, Ag – age groups

	Total	Sex		Age group		
	All *n* = 159 [n/%]	Female *n* = 93 [n/%]	Male *n* = 66 [n/%]	Ag 1 (35–44 yrs) *n* = 50 [n/%]	Ag 2 (65–74 yrs) *n* = 57 [n/%]	Ag 3 (> 75 yrs) *n* = 52 [n/%]
What is your general opinion about oral implants? (Multiple answers possible)						
	*n* = 226	*n* = 134	*n* = 92	*n* = 68	*n* = 79	*n* = 79
I would get implants if needed.	55 / 24.3	31 / 23.1	24 / 26.1	19 / 27.9	22 / 27.8	14 / 17.7
Implants are expensive.	51 / 22.6	31 / 23.1	20 / 21.7	20 / 29.4	14 / 17.7	17 / 21.5
I would prefer not to get implants.	38 / 16.8	24 / 17.9	14 / 15.2	3 / 4.4	9 / 11.3	26 / 32.9
Others.	34 / 15.0	21 / 15.7	13 / 14.1	10 / 14.7	16 / 20.3	8 / 10.1
Implants are not suitable for everyone.	22 / 9.7	13 / 9.7	9 / 9.8	5 / 7.4	7 / 8.9	10 / 12.7
No opinion on this.	14 / 6.2	10 / 7.5	4 / 4.4	7 / 10.3	5 / 6.4	2 / 2.5
Implants are suitable for everyone.	12 / 5.4	4 / 3.0	8 / 8.7	4 / 5.9	6 / 7.6	2 / 2.5
Implants are affordable for everyone.	0 / 0	0 / 0	0 / 0	0 / 0	0 / 0	0 / 0
What are the advantages of oral implants? (Multiple answers possible)						
	*n* = 261	*n* = 149	*n* = 112	*n* = 93	*n* = 91	*n* = 77
Aesthetics	43 /16.5	26 / 17.4	17 / 15.2	20 / 21.5	14 / 15.4	9 / 11.7
Others	36 / 13.8	24 / 16.1	12 / 10.7	11 / 11.8	16 / 17.6	9 / 11.7
Does not feel like removable dentures.	32 / 12.3	20 / 13.4	12 / 10.7	6 / 6.5	14 / 15.4	12 / 15.6
Functionality	30 / 11.5	18 / 12.1	12 / 10.7	12 / 12.9	9 / 9.9	9 / 11.7
No opinion on this.	28 /10.7	15 / 10.1	13 / 11.6	8 / 8.6	7 / 7.7	13 / 16.9
Longer durability/longevity.	26 /10.0	12 / 8.1	14 / 12.5	10 / 10.8	8 / 8.7	8 / 10.4
Comfort Increased bite force. Security in wearing.	22 / 8.4 22 / 8.4 22 / 8.4	14 / 9.4 11 / 7.4 9 / 6.0	8 / 7.2 11 / 9.8 13 / 11.6	7 / 7.5 11 / 11.8 8 / 8.6	7 / 7.7 7 / 7.7 9 / 9.9	8 / 10.4 4 / 5.1 5 / 6.5
What are the disadvantages of oral implants? (Multiple answers possible)						
	*n* = 229	*n* = 135	*n* = 94	*n* = 71	*n* = 87	*n* = 71
High cost	71 / 31.0	41 / 30.4	30 / 31.9	29 / 40.0	22 / 25.3	20 / 28.2
Complications	39 / 17.0	23 / 17.0	16 / 17.0	10 / 14.1	20 / 23.0	9 / 12.7
Surgical procedure	36 / 15.7	23 / 17.0	13 / 13.8	13 / 18.3	12 / 13.8	11 / 15.5
No opinion on this.	33 / 14.4	19 / 14.1	14 / 14.9	9 / 12.7	10 / 11.5	14 / 19.7
Long time until functional	25 / 10.9	13 / 9.6	12 / 12.8	6 / 8.5	12 / 13.8	7 / 9.9
Others	18 / 7.9	12 / 8.9	6 / 6.4	4 / 5.6	9 / 10.3	5 / 7.0
Do not last long	7 / 3.1	4 / 3.0	3 / 3.2	0 / 0	2 / 2.3	5 / 7.0

## Discussion

The hypothesis that the level of knowledge about oral implants is low in all age groups could be confirmed. The hypothesis that knowledge about oral implants decreases with age could not be confirmed. The level of knowledge about oral implants does not correlate with the age of the participants.

### Study design

The study was conducted through telephone surveys. Unlike face-to-face interviews or written dialogues, the monetary and personnel requirements for conducting telephone surveys are relatively low [21]. The response rate from the total sample is low (response rate: ag 1: 12.5%; ag 2: 14.5%; ag 3: 12.8%) compared to alternative methods of data collection (30–40%), although some study participants could not be reached by phone [22]. The adherence and compliance of study participants are lower in telephone surveys compared to face-to-face interviews. Participation is more frequently declined than in personal conversations. The response rate in face-to-face interviews averages 50–70% [22]. However, other research findings in the literature also suggest that it is not the data collection mode per se that influences the response rate, but rather that the response rate depends significantly on the specific combination of target population, salience of the research topic, and data collection mode [23–26]. Face-to-face interviews with older people are often difficult to organize. Older people are reluctant to leave their homes as they become frailer, but they are also reluctant to let strangers (interviewers) into their homes because they are worried that people with criminal energy will come.

The advantages of the chosen survey method in the present study are: The presence of participants at a study center is not necessary. Any uncertainties can be clarified directly during the interview. There is no need to wait for the postal return of the questionnaire. Special software required for computer-assisted surveys is not necessary. Compatibility problems in data transfer are thus excluded. Disadvantages of the data collection method include: Telephone numbers from registration records quickly become outdated due to moves or new connections. Moreover, traditional landline connections are increasingly becoming obsolete. In Germany, mobile phone numbers are not captured in registration records. The primary disadvantage of telephone interviews lies in the limited capacity for information processing and concentration of the participants [27]. Telephone surveys have a higher potential for fatigue compared to other data collection methods. In this regard, the recency effect is of crucial importance. In closed-ended questions, short-term memory assigns greater weight to the most recently mentioned response options, which are consequently mentioned more frequently [28]. The primacy effect in such a survey is also described in the literature. It is assumed that the response options mentioned at the beginning are stored in long-term memory and are therefore easier to recall [29]. Both phenomena cannot be reliably ruled out in the present study. For open-ended questions also posed to the participants, socially desirable responses influence the reliability of the collected data. The comparability of answers to open-ended questions is significantly more complex. This was facilitated by the use of the Likert scale. The Likert scale both simplifies the statistical analysis and reduces the objectivity of the given response [30]. The conscious or unconscious influence by the interviewer conducting the telephone survey also introduces response bias. This interviewer effect can potentially even lead to the phenomenon of reactance, resulting in defensive reactions and refusal to participate in the study. Response tendencies bias is considered a typical methodological problem of questionnaires [31]. It should also be noted that the 67-item questionnaire used was very long, which may have led to concentration problems or fatigue on the part of the participants, possibly influencing the answers. Households without a telephone connection were excluded from the study. Therefore, the generated quota sample is only considered partially representative. As a possible solution strategy, the application of random digit dialing, which involves computer-assisted random generation of telephone numbers, can be mentioned [32].

For this study, the questionnaire was specifically developed and tested for comprehensibility etc. in advance as part of a pilot test with some participants. Validity and reliability were not tested. This should be considered for further studies on the topic. It would also be desirable to reduce the length of the questionnaire.

## Results

### Awareness of implants

In the literature, there is limited data directly correlating the popularity of implants with the age of study participants [33]. In an American study, awareness of artificial tooth roots was highest among those over 60 years old [16]. In another study, age over 50 years was defined as a predictor of higher awareness. The participants at the upper and lower ends of the age scale, i.e., students and retirees, were among those least aware of oral implants [33]. An explanation for this could be that older individuals might have less access to alternative sources of information (e.g., the Internet) to learn about what implants are. Young people are often fully toothed and don’t need to deal with the replacement of lost teeth.

The findings of a Norwegian study showed a similar trend. Oral implants were least known to participants in the age groups of 16–24 and over 80 years old [34]. Alajlan et al. and Siddique et al. describe a similarly high level of awareness of oral implants in the population (Siddique et al.: India 93.4%, Alajlan et al.: Saudi Arabia 91.5%) [35, 36]. In contrast, a study by Khosya et al. reported an awareness rate of 40.4% (*n* = 114). Awareness was highest among the 15–30 age group (44.7%) [37]. Gupta et al. found a popularity rate of 21% (*n* = 400) [38]. This is likely due to the lower educational level of the participants compared to the studies by Zimmer et al., Berge et al., and Pommer et al. [16, 34, 39]. According to Elani et al., the importance of oral implants in the population has been gradually increasing across all age groups, as reflected in the prevalence of oral implants (1999–2000: 0.7%, 2015–2016: 5.7%). The authors even anticipate that the prevalence of oral implant use could rise to 23% by 2026 [1].

The increasing prevalence of oral implants leads to discussions on this topic among friends and acquaintances. Dentists are cited as the primary professional source of information (45.5%), followed by electronic media [36]. This aligns with the results of Awooda et al. (38.2%), Al-Johny et al. (31.5%), Suwal (30.2%), et al. [40–42]. In the present study, age groups 1 and 3, in particular, obtain information about oral implants from friends and acquaintances. In Zimmer et al.‘s study, media and friends also played a role in information acquisition (77%) [16].

The increasing digitization has revolutionized access to information. A study by the German Center for Ageing Issues found that in 2020, 86% of people aged 46 to 90 had private internet access. Between 2017 and 2020, the proportion of people with internet access increased by about 4% points, from 82% in 2017 to 86% in 2020. The increase was most pronounced in the age group of 61 to 75-year-olds [43]. This highlights why awareness of oral implants naturally increases with age.

Regardless of access to the digital world, the dentist holds the greatest significance in information acquisition about oral implants (Esfahani et al.: 40.7%, Kohli et al.: 53.6%, Tomruk et al.: 44.5%, Siddique et al.: 93.4%) [36, 44–46].

### Level of information

In all age groups, study participants feel less informed about oral implants compared to other options for dental prosthetics. Especially at the upper and lower ends of the age scale, at least half of the respondents feel poorly or are not informed about oral implants. One possible cause is the lack of receptiveness or acute relevance of such information in the respective age groups.

Particularly in age group 3, the feeling of discomfort towards newer therapeutic measures may act as an intrinsic barrier, even though the dentist provides equal information to older patients.

Another reason could be the patient’s lack of understanding of the complexity of implant-supported therapy. Here, the in-depth application of the step-by-step concept of shared decision-making could be a possible solution strategy. It involves an interactive process aiming to reach a mutual agreement based on shared information and equal active participation of the patient and the dentist [47]. The implementation of the participation process would be a win-win situation for both the patient and the dentist. Prioritizing patient autonomy and considering individual preferences in the treatment decision gives the patient a certain level of control, leads to higher adherence, and consequently, better clinical outcomes. Patients take responsibility for the informed treatment decision, thereby relieving the dentist as the sole responsible party [48].

The fact that one-third of the participants in age group 3 also feel poorly or are not informed about other treatment options could be related to feeling “too old” for extensive dental restorations and therefore not worth further consideration. The change in specific needs in old age and also the fact that dentists and relatives discriminate on the basis of age (e.g., they can no longer afford it) seem to play an essential role here [49]. Dentists accompany their patients through very heterogeneous life phases with completely different dental care demands over several decades. To support communication, illustrate treatment concepts, and, above all, increase patients’ knowledge, the use of medical decision aids is conceivable [50, 51]. Decision aids in the form of screening tests are already widespread in some areas of medicine (e.g., Mammography). Decision aids are not yet widespread in dentistry. However, according to the study by Johnson et al., there is evidence that their use facilitates both participatory decision-making and the implementation of evidence-based dentistry. These tools could be available in both paper form and computer-based applications such as apps or interactive websites.

### Source of information

The dentist is the most important source of information on oral implants in all age groups if further information is desired. This finding is consistent with numerous studies that also attribute a key role to dentists in information provision [33, 36, 39, 52–55]. Expert opinion seems to carry the most weight in information acquisition and decision-making in the population. The dentist acts as a valid and objective source of information. However, other studies also found other patient preferences. Multiple sources of implant information have been previously reported. For example, in Jordan, patient information regarding dental implant treatment is often obtained from family and friends, with reference to dentists only when patients need additional information [56]. Kashbour et al. reported that in general, patients rated clinical information sources as trustworthy. Nevertheless, it was clear that patients were not receiving information about their own specific situation, concerns and preferences. This may result in them relying on other general sources of information [57]. This may promote the advantages of implant treatment without taking individual needs and variables into consideration [57]. The use of various information methods such as notice boards, advertisements, social media, and in-person contact with non-professionals may lead to altered conceptions of the necessary procedures [58], and unrealistic expectations about dental implants [59].

Improving dental communication strategies could help counter unrealistic expectations regarding the treatment modality of oral implants. The implementation of social and communication skills is directly related to clinical consequences such as treatment adherence and increased satisfaction for both practitioners and patients [50]. Almost half of older people would like a dentist to have social and empathic skills as their first characteristic [60].

### Knowledge

Most of the participants are aware of oral implants as a treatment option. However, it is surprising that there is a lack of knowledge in this area. Tepper et al. found similar results, with a significant discrepancy between stated awareness and knowledge about oral implants. 40% of the respondents could not correctly identify the location of implant anchorage, such as the jawbone [33]. The general population is not aware that oral implants require specific care and hygiene measures [35, 36, 44, 52, 61]. There is also a knowledge deficit in assessing the longevity of oral implants. More than half of the study participants estimated the lifespan of implants as “lifelong” [36, 61–63].

The present study emphasizes the massive information deficit in the population regarding oral implants as a treatment option. It is necessary to understand patients’ concerns about implant therapy, e.g. due to the long treatment time or possible complications, and to minimize these through continuous patient education, reassurance, and support [58].

There was no proven correlation between age and information deficit in this study. Age does not have a significant effect on the level of knowledge regarding oral implants.

Insufficient knowledge or misinformation leads to unrealistic expectations and erroneous evaluation of oral implants [59]. This is directly correlated with the success or failure of implant therapy [59].

In this regard, the optional use of decision aids could be considered to assist dentists in making a joint decision with the patient regarding the best treatment option. One possible example is a questionnaire about the patient’s symptoms, medical history, and preferences. Another option is the use of visual representations such as 3D models or X-ray images to help the patient better understand the consequences of different treatment options. This allows the patient to make a more informed decision.

Additionally, the dentist could provide brochures or flyers on this topic in their practice. The implementation of low-threshold offerings such as public information events can also be a good way to educate the public about oral implants. To reach an even broader audience, social networks would be another option. Dentists and other experts could publish online content on this topic, reducing any uncertainties regarding planned implant treatments.

### Evaluation

When asked about possible disadvantages of oral implants, the high cost was primarily mentioned, followed by concerns about potential complications, the need for a surgical procedure, and the prolonged treatment time.

This correlates with the results of other studies. In these studies, cost intensity, surgical intervention, and extended treatment duration were also defined as primary disadvantages [14, 16, 33, 36, 39]. The study by Al-Johany also identified “fear of the placement of implants concept” as a factor [41].

A detailed explanation of implant treatment planning and comprehensive patient education could be the key to increasing acceptance of oral implants and their cost. The barrier of “surgical intervention” could also be mitigated through adequate education and prevention of misinformation, which is one of the crucial pillars in patient treatment. However, this should not lead to an overextension of indications. In addition, patients must be informed about implant maintenance. It is known that patient motivation can contribute to reducing mucositis and the risk of inflammation around implants [64].

The triad of therapeutic capability, oral hygiene ability, and self-responsibility within the framework of oral functional capacity can help identify limitations of oral implantology. Evaluating social resources and prospective post-treatment competence are equally important, as are potential comorbidities, especially in elderly patients [65]. Despite the ever-aging population, oral implants remain an indispensable treatment option. In many cases, they improve the quality of care and expand the scope of prosthetic interventions.

## Conclusion

The results of this study help to identify information deficits as intrinsic barriers to treatment with oral implants. Here, representatives at the meso-level of the health care system, e.g., health insurance companies, patient counselling centers, are just as much called upon as the exchange of information in direct individual contact between patient and dentist (micro level). The implementation of new and especially age-appropriate sources of information could support the option of treatment with oral implants. Older people should be given the opportunity to improve their health literacy in oral implant use. Physicians should also be informed about the basics of oral implantology. It is known that patients sometimes also ask their general practitioner about these topics and pay attention to their opinion than of the specialists, the dentists. Targeted and efficient educational programs can be developed based on the collected data.

## Data Availability

The datasets used and/or analyzed during the current study are available from the corresponding author upon reasonable request.
